# Radiological and Morphometric Features of Canalis Sinuosus in Russian Population: Cone-Beam Computed Tomography Study

**DOI:** 10.1155/2019/2453469

**Published:** 2019-12-16

**Authors:** Avanesov Anatoly, Yuri Sedov, Evgeniya Gvozdikova, Oleg Mordanov, Liudmila Kruchinina, Karen Avanesov, Anna Vinogradova, Sergei Golub, Dalila Khaydar, Nguyen Giao Hoang, Hadi M. Darawsheh

**Affiliations:** Department of General and Clinical Dentistry, RUDN University, Medical Institute, Moscow, Russia

## Abstract

**Introduction:**

Cone-beam computed tomography (CBCT) is considered to be the most informative radiographic method for pre- and postoperative analysis of the maxillary anatomy and for avoiding further complication. Canalis sinuosus is one of such structures that damage can go along with bleeding and neurological symptomatology. The aim of the study was to investigate radiological and morphometric features of the canalis sinuosus in Russian population using CBCT technique.

**Materials and Methods:**

150 CBCT scans of 61 males and 89 females aged from 24 to 80 years were retrospectively studied with different slice thickness and evaluated with regards to prevalence and diameter among age and gender groups in Russia.

**Results:**

CS prevalence in this study was 67%, and CS was most frequently presented in the lateral incisor region (33.5%). Women showed statistically higher CS prevalence (*p* < 0.01) than the male group, and there was no statistically significant difference observed between occurrence and localization of CS and age groups.

**Conclusion:**

CBCT examination demonstrated good diagnostic efficiency in CS visualization, and the CS may have variations on its location and prevalence with statistically significant differences between the gender group and without significant differences among age groups and can depend on the population.

## 1. Introduction

Dental implant placement in the anterior maxilla is a medium or high complexity challenge and requires careful preoperative planning [1]. Two-dimensional radiographic techniques have multiple limitations for such cases that is why cone-beam computed tomography (CBCT) is considered to be the most informative radiographic method for performing comprehensive pre- and postoperative analysis of the maxillary anatomy and, consequently, for avoiding further complication [2–5].

The significant variability in maxillary neovascularization and high trabecular anterior maxilla density emphasizes the importance of three-dimensional diagnostics and avoiding this method poses a risk for bleeding and neurological symptomatology [6, 7].

One of such structures, that damage can go along with these complications, is canalis sinuosus (CS) [8]. CS is an intrabony structure that carries the anterior superior alveolar nerve and vessels. For example, Machado et al. [9] presented two case reports where patients suffered from pain and it immediately relieved after implant extractions that were placed with CS damaging.

The recent systematic literature review of CS [6] studies showed that the terminal portion of CS is more prevalent in the anterior region of the maxilla, more specifically in the incisor and canine region near the palate [10–14], and that locations can be favourable positions for better dental implant anchoring and esthetic outcomes in the anterior maxilla [1]. However, as the literature review showed there is no CBCT or other studies of CS in Russia where dental practitioners still use the data obtained from foreign authors.

The aim of this study was to investigate radiological and morphometric features of the canalis sinuosus in Russian population using CBCT technique.

## 2. Materials and Methods

150 CBCT scans of 61 males and 89 females aged from 24 to 80 years (mean age is 63.27 ± 6.8) with 10 × 8.5 field of view were retrospectively studied, who attended the radiologic diagnostic center for three-dimensional radiological scanning for different diagnostic purposes. Written consent was signed by all individuals before taking the procedure. The CBCT device had the following characteristics: 0.2 mm/0.3 mm voxel size; 0.5 mm focal spot; 18 sec scanning time; and 55–99 kB/4–16 mA tube voltage.

The following exclusion criteria were used: the presence of a supernumerary or retained tooth in the anterior maxilla, the presence of a pathological or traumatic lesion in this region, patients who had previously undergone a surgical procedure in the maxilla, and the presence of technical artefacts.

Firstly, the scans were analyzed in Ez3D Plus (Vatech Co., Korea, 2009), software on panoramic and cross-sectional views with 0.5 mm, 1 mm, 3 mm, and 10 mm slice thicknesses. CS was identified according to its description in the literature [9].

Secondly, mesiodistal location according to the Oliveira-Santos et al. classification [15] and facial-palatal location were determined. The prevalence and location were analyzed with regards to age: young group (24–44 years), middle age (45–59 years), and elderly (60 years and more) and gender groups using one-way ANOVA test with StatPlus 6 (AnalystSoft).

## 3. Results

**Figure 1:**
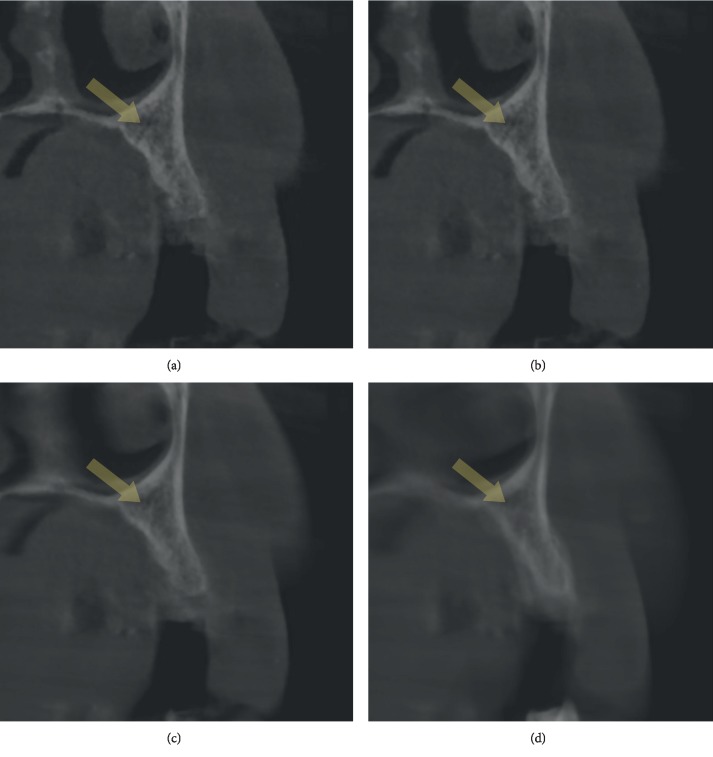
CS with different slice thicknesses in the same patient: (a) 0.5 mm, (b) 1 mm, (c) 3 mm, and (d) 10 mm. Note that CS is almost visualized with 3 mm and 10 mm slice thicknesses.

The slice thickness of CBCT scans was sequentially changed from 0.5 mm to 1 mm to 3 mm and to 10 mm. CS on images with different slice thicknesses was evaluated separately (Figure 1).

**Figure 2:**
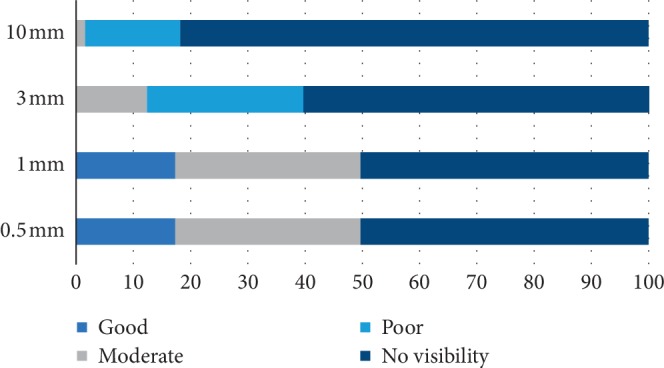
CS visualization with the four-point rating scale: no visibility—important structures are not visualized; poor—important structures are not diagnostic; moderate—important structures are diagnostic but could be improved; good—important structures are optimally visualized.

Then, CS visualization was graded with the four-point rating scale. This method was precisely and comprehensively described by Jacobs et al. [16] while evaluating the mandibular incisive canal with CBCT. This method showed that the optimal visualization of CS was reached with 0.5 mm/1 mm slice thickness (Figure 2).

Evaluating CBCT scans with 1 mm slice thickness, the alveolar process part of CS was evident in 101 of 150 patients (67% of total patients). 22 of these 101 patients (21.7%) presented with CS only on the right side, 33 (32.6%) patients only on the left side, and 47 (45.7%) patients on both sides. In total, 149 CS were identified on both sides with 0.5 mm slice thickness and CS was presented in 46% on the right side (22 + 47 of total 149 canals) and 54% on the left side (32 + 47 of total 149 canals) and this difference was not statistical (*p*=0.6).

Data on CS localization according to Oliveira-Santos et al. and facial-palatal localization are shown in Tables 1 and 2 consequently. Most often CS was located in the lateral incisor region and palatally. In addition, statistically more (*p* < 0.01) female persons presented CS compared to the male group. However, there was no statistically significant difference between age groups (*p*=0.8).

## 4. Discussion

It is extremely important that the professional has knowledge about the trajectory and caliber of the CS, aiming at the prevention of injury during dental procedures that surround it [6]. Several studies and clinical cases showed that CBCT is the best radiographic technique for CS visualization [6]. CBCT application is recommended to identify CS and understand its location, diameter, length, and variation, avoiding possible iatrogenic complications in the implant site or other surgical procedures involving the anterior maxilla [17].

However, several studies demonstrated different conclusions on the dependence of the slice thickness of CBCT scans and the detection of the anatomical and other structures, as well as the way that reduces artefacts [18–24]. Our choice of slice thickness was 1 mm for good and moderate detection of CS without missing it and with high reduction of artefacts in this case.

Ferlin et al. [6] showed that the prevalence of CS in different populations using different study methods ranged from 52.1% to 100% of samples. Our study of Russian population revealed 67% of CS prevalence. This prevalence was close to a German study (67.6%) by Ghandourah et al. [13] and Turkish study (70.8%) by Orhan et al. [14].

Similar to other studies, this study of Russian population showed that there was no statistically significant difference with regards to the side. In addition, CS was most frequently presented in the lateral incisor region (33.5%), central incisor region (24.2%), and canine region (21.5%) and near the palate that makes its localization regarding to different directions close to other populations [6].

Gurler et al. [10] and von Arx et al. [11] noted that men showed a higher prevalence in relation to CS, but without statistically significant differences, as well as Machado et al. [9] who found an increased prevalence of males over females, but in our Russian population the study women showed statistically higher CS prevalence (*p* < 0.01) than the male group with the same tendency of CS localization described above.

There was no statistically significant difference observed between occurrence and localization of CS and age groups. Orhan et al. [14] in the Turkish group and Ghandourah et al. [13] in the German population observed higher prevalence of CS in older age groups in comparison with young adults, and our study showed higher prevalence in the young group (32%).

## 5. Conclusion

CBCT examination demonstrated good diagnostic efficiency in CS visualization (67%). In addition, this study showed the importance of slice thickness choice for CS visualization. The best visualization was reached with 0.5 mm and 1 mm slice thicknesses. The results of the Russian population study allow us to conclude that the CS may have variations on its location and prevalence with statistically significant differences between the gender group and without significant differences among age groups and can depend on the particular population.

## Figures and Tables

**Table 1:** CS localization according to Oliveira-Santos et al.

No	Region	Total % (*n*)	Gender % (*n*)		Age % (*n*)		
			Male	Female	24–44	45–60	60 and more
1	Central incisor	24.2 (36)	8.7 (13)	15.4 (23)	7.3 (11)	6.7 (10)	(15)
2	Between central and lateral incisors	10.7 (16)	5.3 (8)	5.3 (8)	4 (6)	2 (3)	(7)
3	Lateral incisor	33.5 (50)	13.4 (20)	20.1 (30)	10.7 (16)	10 (15)	(19)
4	Canine	21.5 (32)	9.3 (14)	12 (18)	11.4 (17)	4.6 (7)	(8)
5	First premolar	9.4 (14)	2.6 (4)	6.7 (10)	6 (9)	2 (3)	1.3 (2)
6	Lateral to incisive foramen	0.7 (1)	0.7 (1)	0	0.7 (1)	0	0
7	Posterior to incisive foramen	0 (0)	0	0	0	0	0
			40.2 (60)	59.8 (89)	40.2 (60)	25.5 (38)	34.3 (51)

**Table 2:** CS localization regarding to facial-palatal position.

No	Position	Total % (*n*)	Gender % (*n*)		Age % (*n*)		
			Male	Female	24–44	45–60	60 and more
1	Facial	12 (18)	4 (6)	8 (12)	0.7 (1)	5.3 (8)	6.1 (9)
2	Central	12 (18)	6.7 (9)	5.3 (9)	7.3 (11)	4.1 (6)	0
3	Palatal	76 (113)	30 (45)	46 (68)	32 (48)	16.7 (25)	26.8 (40)

## Data Availability

The data used to support the findings of this study are included within the article.
